# Warm plankton soup and red herrings: calcareous nannoplankton cellular communities and the Palaeocene–Eocene Thermal Maximum

**DOI:** 10.1098/rsta.2017.0075

**Published:** 2018-09-03

**Authors:** Samantha J. Gibbs, Rosie M. Sheward, Paul R. Bown, Alex J. Poulton, Sarah A. Alvarez

**Affiliations:** 1 Ocean and Earth Sciences, National Oceanography Centre, Southampton, University of Southampton, Southampton SO14 3ZH, UK; 2 Department of Earth Sciences, University College London, Gower Street, London WC1E 6BT, UK; 3 National Oceanography Centre, Southampton SO14 3ZH, UK; 4 School of Geographical Sciences, University of Bristol, University Road, Bristol BS8 1SS, UK

**Keywords:** Palaeocene–Eocene Thermal Maximum, calcareous nannoplankton, palaeobiology, shelf, oligotrophic gyres, feedback

## Abstract

Past global warming events such as the Palaeocene–Eocene Thermal Maximum (PETM—56 Ma) are attributed to the release of vast amounts of carbon into the ocean, atmosphere and biosphere with recovery ascribed to a combination of silicate weathering and organic carbon burial. The phytoplanktonic nannoplankton are major contributors of organic and inorganic carbon but their role in this recovery process remains poorly understood and complicated by their contribution to marine calcification. Biocalcification is implicated not only in long-term carbon burial but also both short-term positive and negative climatic feedbacks associated with seawater buffering and responses to ocean acidification. Here, we use exceptional records of preserved fossil coccospheres to reconstruct cell size distribution, biomass production (particulate organic carbon, POC) and (particulate) inorganic carbon (PIC) yields of three contrasting nannoplankton communities (Bass River—outer shelf, Maud Rise—uppermost bathyal, Shatsky Rise—open ocean) through the PETM onset and recovery. Each of the sites shows contrasting community responses across the PETM as a function of their taxic composition and total community biomass. Our results indicate that nannoplankton PIC:POC had no role in short-term climate feedback and, as such, their importance as a source of CO_2_ to the environment is a red herring. It is nevertheless likely that shifts to greater numbers of smaller cells at the shelf site in particular led to greater carbon transfer efficiency, and that nannoplankton productivity and export across the shelves had a significant modulating effect on carbon sequestration during the PETM recovery.

This article is part of a discussion meeting issue ‘Hyperthermals: rapid and extreme global warming in our geological past’.

## 1. Introduction

Past transient global-warming events such as the Palaeocene–Eocene Thermal Maximum (PETM) approximately 56 million years ago are attributed to the release of vast amounts of carbon into the ocean, atmosphere and biosphere with the recovery of the Earth system largely ascribed to increased weathering of silicates and/or increased rates of organic carbon burial [1,2]. However, the relative contributions of these feedback mechanisms and how and when they operated within the tens to hundreds of thousands of years that followed the onset of these events are still poorly constrained [1,3]. Evidence is primarily drawn from biogenic carbon, carbonate and barite accumulation rates, carbon isotopes, palaeoecological trends and Earth system models [1–4]. The biotic proxies rely heavily on the robust and ubiquitous fossil records of calcareous nannoplankton (predominantly coccolithophores), planktonic and benthic foraminifera, and dinocysts. Despite some incongruities, the analysis of assemblage compositions and abundances has led to broad agreement on the marine response to the PETM, with increased productivity in coastal and continental margin regions but decreased productivity in the open ocean [4–8]. Palaeoecological analysis of nannoplankton has unpinned documentation of the spatial heterogeneity of biotic response at the PETM [4,7–12] but there have also been attempts to quantify rates of production/export through the event [4], and to uncover evidence of disruption of calcification brought on by changing atmospheric and ocean chemistry [4,13–15]. These endeavours are challenging given the potential biases in the fossil record, which become particularly acute during the PETM, as carbonate dissolution caused widespread modification of the preserved record and introduced uncertainties regarding carbonate production versus preservation [4]. Furthermore, the specific role of calcareous nannoplankton in any productivity-feedback on climate remains elusive because speculation surrounds the duplicity of nannoplankton calcification, with its theoretical counteracting effect on the short-term buffering capacity of surface waters (e.g. [16–21]), and whether this might have acted to reduce the effectiveness of plankton involvement in atmospheric CO_2_ drawdown during the event. One avenue that has remained largely unexplored, however, is the extraction of detailed information on the cellular characteristics of these ancient plankton and how cells and communities of cells varied in space and time through these intervals of tumultuous environmental change. In particular, we have yet to document cell-size distributions or ratios of organic to inorganic carbon across ancient nannoplankton species and communities. As such, we currently have little to no appreciation of the calcareous nannoplankton contribution to carbon sequestration during extreme transient events like the PETM that we know are characterized by large shifts in assemblage composition. Here, we take advantage of exceptionally preserved nannoplankton records with unusually abundant occurrences of entire exoskeletons (coccospheres) that allow us to make direct measurements of cellular traits, such as cell volume and calcite mass, and, for the first time, to quantify cell-size frequency distributions across communities through the PETM event. Further, we extrapolate from these unique data to reconstruct population biomass for three distinct oceanographic settings (shelf, off-shelf and open ocean), assessing the impact of climate change on these cellular characteristics and the significance of the changes for carbon sequestration and the Earth system feedbacks which operated during the recovery.

## 2. Material and methods

### (a) Material

**Figure 1.**
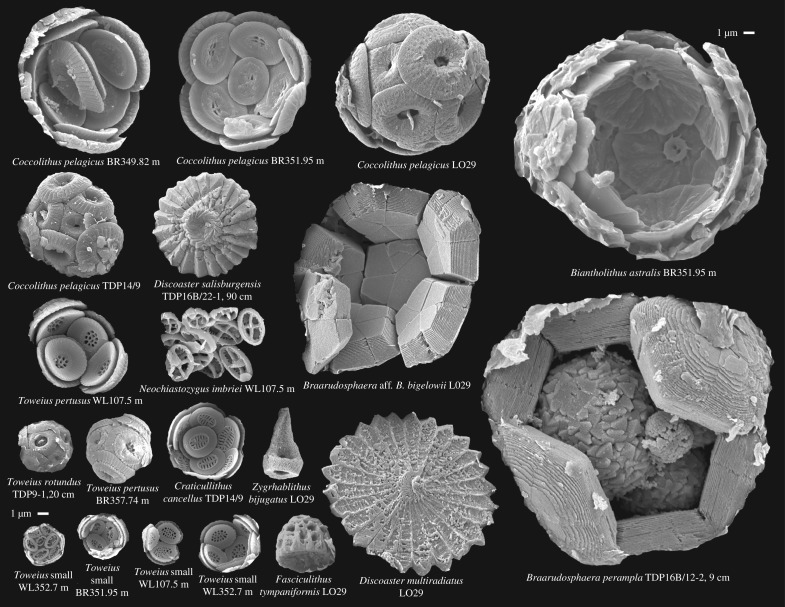
A representative selection of PETM coccosphere types and sizes, all shown on the same scale (scale bars are 1 µm). Also included are examples of a collapsed murolith coccosphere of *Neochiastozygus imbriei*, a disarticulated holococcolith of *Zygrhablithus bijugatus* and nannoliths of *Discoaster salisburgensis* and *D. multiradiatus* and *Fasciculithus tympaniformis*. Sample prefixes: WL—Wilson Lake, BR—Bass River, LO—Lodo Gulch, TDP—Tanzania Drilling Project.

Our analytical approach (see §2c) requires the integration of PETM coccosphere measurements with fossil calcareous nannoplankton (‘nannofossil’) assemblage data. Fossil coccospheres allow us to document cell size, coccosphere geometry and exoskeletal calcite mass, and assemblage data provide relative abundance distributions of nannoplankton communities across both space and time. Preservation of coccospheres is not typical in nannofossil records and requires targeting samples that satisfy a number of taphonomic requirements, usually including clay-rich host sediments, shallow burial-depths and low bioturbation-intensity [22]. Here our coccosphere data come from sites with exceptional nannofossil preservation, including Bass River and Wilson Lake (New Jersey), Lodo and Tumey Gulches (California), ODP Site 401 (Bay of Biscay), and Kilwa (Tanzania) [22] (examples in figure 1). High numbers of PETM-interval coccospheres were present at all these sites and some data were previously presented in [23,24]. Our (published) assemblage data come from three sites that lie in distinctly different settings of the PETM marine realm: Bass River, a mid-latitude, high-productivity shelf setting off the North American seaboard (onshore drillsite, ODP Leg 174AX [4]); Maud Rise, an off-shelf, high-latitude site in the south Atlantic sector of the Southern Ocean (ODP Site 690 [4]); and Shatsky Rise, an open-ocean, central gyre, low-latitude site in the Pacific (ODP Site 1209 [4,7]). For comparative purposes, the assemblage data are grouped into three key time-slices relative to the stratigraphy of the carbon isotope excursion (CIE) that defines the PETM [25–27]—the pre-CIE (the averaged assemblage composition from immediately below the first expression of the carbon isotope excursion), the peak of the PETM event (averaged assemblage composition across samples from within the core of the CIE before isotopic levels began to increase into the recovery interval), and the PETM recovery interval (averaged assemblage composition across samples within the core of the recovery phase where carbon isotope values were returning to, but had not yet reached, a stable post-event level).

### (b) Fossil coccospheres, cell geometries and estimates of cellular PIC and POC levels

Fossil coccospheres were imaged from simple smear slides [28] using light microscopy at ×1000 magnification following the imaging and measurement procedures of [23] and [29]. Two images were taken of each coccosphere, one focused on the maximum outer coccosphere circumference, allowing for the coccosphere and cell dimensions to be measured (the internal dimension representing the position of the original cell), and the second focused on the proximal surface of the coccolith tube-cycle of a representative coccolith on the coccosphere surface, allowing for the coccolith dimensions to be measured. We collected additional disarticulated coccolith size data for each taxon from the same samples in order to determine the complete range of coccolith sizes present in each assemblage. As cell size and coccosphere size typically vary proportionally with coccolith dimensions [23,29,30], this enabled us to incorporate cell sizes that may not have been fully represented in the preserved coccosphere record (see §2c). We have supplemented these light microscope data with measurements and general observations from scanning electron microscope (SEM) imaging of rock-chip surfaces that allow *in situ* observations of nannofossils and coccospheres (see [31]).

The fossil record of coccospheres is dominated by coccolithophore taxa that form placolith-type coccoliths, which physically overlap and interlock to form a mechanically robust covering around the cell [22,23,30]. These placolith taxa are also often numerically dominant in nannoplankton assemblages and so for the majority of the PETM taxa we have direct coccosphere measurements. The remaining nannoplankton groups present at this time form murolith coccoliths (disc-like morphologies), holococcoliths (often broadly disc-like) and nannoliths (non-coccolith morphologies), which lie side-by-side to form coccospheres structurally-bound by organic materials [22]. These taxa rarely or never survive as coccospheres in the fossil record and so we rely on modern analogue species, the observation of collapsed coccospheres, and/or geometric considerations in order to reconstruct coccosphere and cellular attributes (see electronic supplementary material). One exception to this is the Braarudosphaeraceae group whose coccospheres relatively frequently occur as fossils and for which we have direct measurements. The only common PETM holococcolith is the unusually large and robust species *Zygrhablithus bijugatus* and we have observed several collapsed coccospheres that have guided our coccosphere reconstructions. The PETM nannolith groups are dominated by *Discoaster*, *Fasciculithus* and *Sphenolithus*, all of which are extinct and have no appropriate extant analogue species. We have made the necessary assumptions that there are fundamental constraints on cell geometry imposed by nannolith morphology (especially lith curvature) and that most cells would have been spherical or sub-spherical as this is the most common cell shape across extant (and fossil) coccolithophores. Together, these direct coccosphere measurements and reconstructions allow us to document cell size (*Θ*), the taxon-specific relationship (the ‘geometry’) between number of coccoliths per cell (*C*_N_), coccolith length (*C*_L_) and *Θ* (table 1), and to calculate particulate organic carbon (POC) per cell and particulate inorganic carbon (PIC) per cell from these parameters (see electronic supplementary material).

**Table 1.** Summary of main biometric parameters measured or reconstructed from PETM coccospheres and loose coccoliths/nannoliths. *C*_N_ is the number of coccoliths per cell, *Θ* is cell diameter, and *C*_L_ is coccolith length.

taxon	lith type	mean *C*_N_	*C*_N_ range	*Θ* range (µm)	*C*_L_ range (µm)^a^	geometry (power law relationship)^b^	shape factor^c^	
*Coccolithus*	placolith	12.9	6–23	3.3–18.2	2.1–12.9	*α* = 1.722, *β* = 0.417	0.06	using modern *Coccolithus*
*Toweius*	placolith	7.2	5–14	2.7–12.1	1.6–8.3	*α* = 1.664, *β* = 0.418	0.055	assuming slightly less calcified than *Coccolithus*
small *Toweius*	placolith	11.5	6–22	2.0–5.6	1.5–4.7	*α* = 1.664, *β* = 0.418	0.055	assuming slightly less calcified than *Coccolithus*
*Cruciplacolithus* and *Campylosphaera*	placolith	14	8–25	4.3–12.9	3.8–8.7	*α* = 1.347, *β* = 0.408	0.03	assuming significantly less calcified than *Coccolithus*
*Cyclicargolithus*	placolith	11.7	10–13	5.8–8.0	4.3–5.6	*α* = 1.909, *β* = 0.346	0.08	using *Calcidiscus leptoporus*
*Markalius*	placolith	12	10–14	7.6–17.9	5.4–16.1	*α* = 0.580, *β* = 0.802	0.07	assuming slightly more calcified than *Coccolithus*
*Umbilicosphaera*	placolith	6.6	6–9	2.9–4.8	2.9–5.0	too few coccospheres	0.015	using *Neosphaera coccolithomorpha*
*Chiasmolithus*	placolith	8.3	6–12	8.5–16.6	3.8–17.2	*α* = 1.608, *β* = 0.445	0.06	using modern *Coccolithus*
*Biscutum*	placolith	19.5	14–25	12.1–20.8 (long axis)	4.9–7.2	*α* = 3.3815, *β* = 0.1916	0.03	assuming significantly less calcified than *Coccolithus*; note that power law relationship less robust because cells are elongate and lith length less directly correlated with cell size.
*Biantholithus*	placolith	13	only 3 specimens	12.3–15.4	11.1–14.1	too few coccospheres	0.07	assuming slightly more calcified than *Coccolithus*
*Braarudosphaera*	nannolith	12	12	13.1–23.0	6.5–15.3	*α* = 1.780, *β* = 0.464	n.a.	direct measurements from coccospheres/liths
*Fasciculithus* and *Sphenolithus*	nannolith	42.5	27–60*	14.0–29.4***	2.8–6.4 (base diameter)	*Θ* = 4.25*C*_L_ + 2.18*	0.6–0.8	based on estimates herein of percentage space within a cone
*Discoaster*	nannolith	10.2	20–31*	5.1–26.1***	3.5–17.9 (rosette diameter)	*Θ* = 1.93C*_L_	0.04	using a ‘rosette’ morphology; shape factor based on half modern *Calcidiscus*
Muroliths	murolith	32.5	20–45**	9.7–25.6***	4.3–11.7	*Θ* = 2.13*C*_L_ + 0.48*	0.035	based on volume calculation herein
*Zygrhablithus bijugatus*	holococcolith	32	32*	8.1–20.8***	2.9–7.4 (base diameter)	*Θ* = 2.8C*_L_	n.a.	using volume calculation herein of a simple base with a hollow, medium-sized spine

*Θ and *C*_N_ are from reconstructed coccospheres which for **murolith-bearing coccospherers are reconstructed based on modern analogues. ^a^Lith size range is based on measurements of loose coccoliths in the same samples as where the coccospheres were found. ^b^The calculation of cell size from coccosphere geometry takes the form *C*_L_ = *α*(cell surface area/*C*_N_)^*β*^. ***Geometries are simplified for murolith and nannolith-bearing taxa and assume a constant *C*_N_. ^c^Shape factors (*k*_s_) are after [32] unless otherwise stated and are used to calculate cellular PIC via the equation 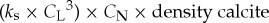 (ref. [32]).

### (c) Reconstructing community cell size distribution and biomass

The distribution of cell sizes within a nannoplankton community is dependent on the frequency distribution of cell sizes within each species and the relative abundance of each of these species. Therefore, in order to reconstruct the cell-size frequency distribution of nannoplankton communities at our three sites (Bass River, Shatsky Rise, Maud Rise) we (i) derived the frequency distribution of cell sizes within each taxonomic group; (ii) normalized each cell-size histogram to the relative cell abundance of each taxonomic group; and then (iii) combined the abundance-normalized histograms of each taxonomic group to generate the cumulative cell-size frequency distribution for the total community.

**Figure 2.**
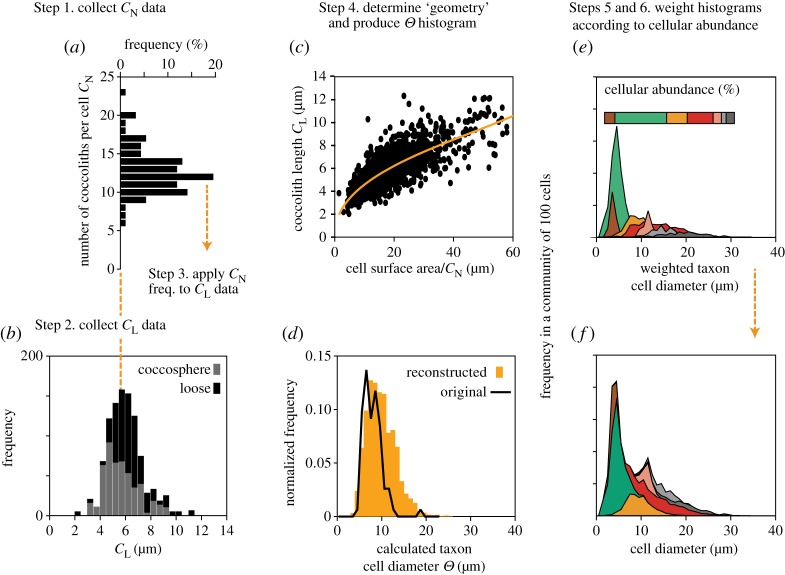
Illustrative schematic of the method used to reconstruct the frequency distribution of taxon cell size within each interval using fossil coccosphere geometry data. Firstly, the cell size distribution for each taxon is reconstructed based on measured fossil coccosphere geometry (*a*–*d*). The frequency distribution of number of coccoliths per cell (*C*_N_) shown in (*a*) is applied to each size bin of the coccolith length (*C*_L_) measured from loose coccoliths in the same assemblage (*b*, black histogram data) to calculate the number of cells of a particular taxon with specific combinations of *C*_N_ and *C*_L_. The cell size of these combinations is calculated using a power-law relationship between cell size (*Θ*), *C*_L_ and *C*_N_ derived from fossil coccosphere measurements (*c*). The abundance of cells in each size class is then compiled to produce a frequency histogram of cell size for this taxon (*d*, orange histogram, as compared to the histogram of only measured fossil coccosphere cell sizes in black). Cell size histograms are then produced in the same way for all other taxa in the community. Site-specific community cell size is then reconstructed by weighting each taxon histogram by their relative cellular abundance in the community during each time interval (*e*) before stacking the frequencies in each size class (*f*) to produce an overall community cell size distribution.

For each taxon the method proceeds as follows (figure 2):

1. Plot the frequency distribution of *C*_N_ from fossil *coccosphere* geometry data (figure 2*a*).
2. Plot the frequency distribution of loose *coccolith* lengths (*C*_L_) from the same samples (figure 2*b*). Measurements from an additional 100–300 loose coccoliths per taxon provide a necessary check on the expected range of cell sizes in each assemblage because preserved coccospheres tend to underrepresent the larger size classes.
3. Apply the *C*_N_ frequency plot across each *C*_L_ size bin, as extensive coccosphere geometry characterization of extant coccolithophores in culture show that the frequency distribution of *C*_N_ is consistent across the range of coccolith lengths [29]. As an illustrative example, imagine that 120 coccoliths in the assemblage fall within the 6.0–6.5 µm coccolith length category (e.g. figure 2*b*), and we know, from our *C*_N_ frequency plots of this taxon that 20% of the coccoliths (i.e. 24 coccoliths) in any *C*_L_ size bin are associated with cells that have 12 coccoliths per cell; this *C*_N_–*C*_L_ combination would represent 2 cells formed of 12 coccoliths that are 6.0–6.5 µm in length. Of the remaining 96 coccoliths, the *C*_N_ histogram tells us that 18 coccoliths (15%) would be associated with cells that have 10 coccoliths per cell, thus representing 1.8 cells, and so on across the remaining *C*_N_ distribution. This is repeated for all of the *C*_L_ size bins until we know how many cells in the population of this taxon have *x* number of coccoliths of *x* µm in length.
4. Calculate the cell size of these *C*_L_–*C*_N_ combinations using the taxon-specific power-law relationship (the ‘geometry’) that exists between *C*_N_, *C*_L_ and *Θ* (figure 2*c*; table 1). This produces a histogram of cell size for each taxon (figure 2*d*). For the non-placolith taxa we applied a more basic geometry, derived using just one value of estimated *C*_N_ but again using measured loose coccoliths.
5. Introduce assemblage data by first converting *lith* per cent abundances into *cell* abundance using average lith number per cell per taxon (table 1).
6. Integrate resultant cell abundance and size distributions by weighting each cell-size histogram by its abundance in the community (figure 2*e*) and stacking the resultant histograms to produce the overall community cell size distribution (figure 2*f*).
7. Transform community cell size histograms into equivalent cell biomass where the area under the curve corresponds to the total biomass of 100 cells (expressed as organic carbon per cell) and the relative position along the axis corresponds to biomass distribution by equivalent cell size. The further to the right along the axis, the greater the proportion of biomass partitioned within larger cells.

### (d) Calibrating nannofossil assemblages and nutrient availability

**Figure 3.**
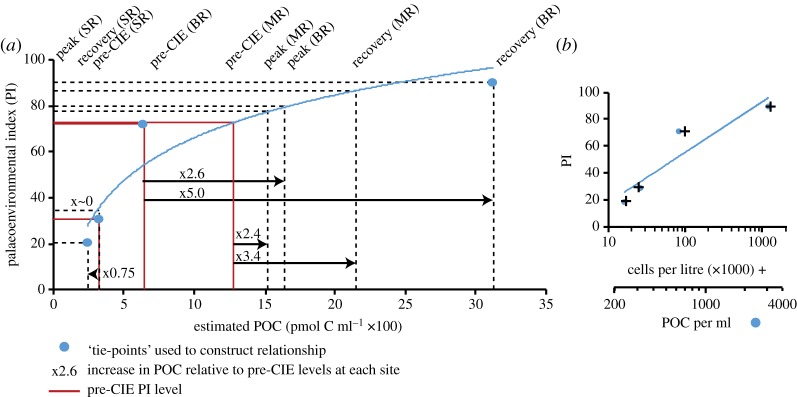
Calibrating the nannofossil-based palaeoenvironmental index (PI) with estimated levels of biomass in seawater, expressed as particulate organic carbon (POC, pmol C ml^−1^). PI is calculated from nannofossil relative abundance data as total mesotrophic/eutrophic water-favouring nannofossil taxa divided by mesotrophic/eutrophic water-favouring taxa plus oligotrophic-favouring taxa with a value approaching 100 indicating dominance by mesotrophic/eutrophic favouring taxa. In (*a*), the red lines indicate the pre-CIE PI and resultant estimated POC values for each site and the times increases in POC for each site are relative to these pre-CIE values. The blue circles highlight the tie-points described in the text to construct the estimated PI to POC relationship. The dashed horizontal lines show the PI values for the non-tie-point time-slices from the three sites and the vertical dashed lines show the resultant estimated POC values. In (*b*) are the PI versus POC tie-points (both shown here on a log scale) with their equivalent cell numbers illustrating the close relationship between POC and cell numbers but demonstrating subtle differences that are because of variations in cell volume between the different tie-points.

Our final step (step 8) scales the biomass plots according to estimated nannoplankton-biomass carrying capacity (effectively the nutrient availability) of the seawater at each site through time. We do this by adjusting the vertical amplitude of the biomass histograms in order to scale the total area under the curves with the relative level of surface-seawater biomass estimated for each site and for each time-slice. First, we place our sites on a common scale according to what the taxic composition of the assemblages tell us about nutrient regime. Specifically, we use the relative abundances of warm-water, oligotrophic-favouring-nannofossil taxa (*Discoaster*, *Fasciulithus*, *Sphenolithus* and *Coccolithus*) versus cooler-water, mesotrophic-favouring taxa (*Toweius* and *Chiasmolithus*) to form a palaeoenvironmental metric (the Palaeoenvironmental Index—PI) (modified from [4]; figure 3). Coccolithophores are strongly responsive to nutrient availability, favouring the same conditions as the majority of plankton, and they typically increase in abundance at the same time of year as increasing nutrients [33–35]. Therefore, we use the PI values as a means of quantifying relative differences in nutrient regime between our sites, with the differences in magnitude of the metric broadly consistent with our understanding of their latitude, nutrient availability and response to the PETM environmental change. This approach is also in general agreement with a previous attempt to scale export productivity changes across the PETM using biogenic barite [3]. We then calibrate our PI by placing estimated levels of nannoplankton standing cell-abundances per litre seawater (converted into biomass and expressed as organic carbon per millilitre of seawater) against our metric using modern shelf and gyre sites as equivalent end-member oceanographic settings. The values we use may not be wholly analogous for the Palaeogene oceans and biota, where, in particular, diatoms were less abundant, but they are a first order estimate of realistic variations. At the lower end of the scale for the pre-CIE PI value at the Shatsky Rise gyre site, we have placed a conservative estimate of 25 cells per millilitre based on measures of 25–35 cells ml^−1^ in modern subtropical gyres [36]; 25 cells ml^−1^, using our Shatsky pre-CIE cell size community structure, corresponds to a total community biomass yield (our estimated nannoplankton carrying capacity) of 313 pmol POC ml^−1^. We then impose an arbitrary reduction of a third of community biomass into the peak PETM, where nannofossil communities indicate reduced nutrient availability caused by warming and stratification [7]. Towards the upper end of the PI scale, we use a conservative 100 cells ml^−1^ for the pre-CIE PI value at Bass River, which corresponds to a community biomass of 630 pmol POC ml^−1^. This is based on normal (non-bloom), coccolithophore cell-counts ranging from 70 to 100 cells ml^−1^ from the temperate, productive shelf-seas off the UK [35]. We then impose a conservative fivefold increase in community biomass reflecting the increased nutrient runoff across the event [7,26], based on the range of chlorophyll *a* measured on the UK shelf from the summer productivity minimum to the spring increase in nutrients, a range of 0.1 to 8.0 mg m^−3^ [37]. This is consistent with accumulation rate estimates of carbonate at Bass River, where rates increased from 0.16 to 1.86 g m^−2^ kyr^−1^ from pre-CIE to the peak of the CIE, approximately an order of magnitude increase, although some increase is due to enhanced carbonate preservation [26]. As PIC:POC in coccolithophores is generally close to 1 (figure 3) we can use carbonate accumulation to infer associated minimum levels of surface water POC. Using these values of community biomass/carrying capacity, the Shatsky Rise and Bass River end-members top and tail the PI calibration and allow us to scale the biomass histograms across the time-slices as well as placing biomass estimates on the intermediate PI values recorded at Maud Rise (figure 3).

## 3. PETM cellular communities and their biomass

**Figure 4.**
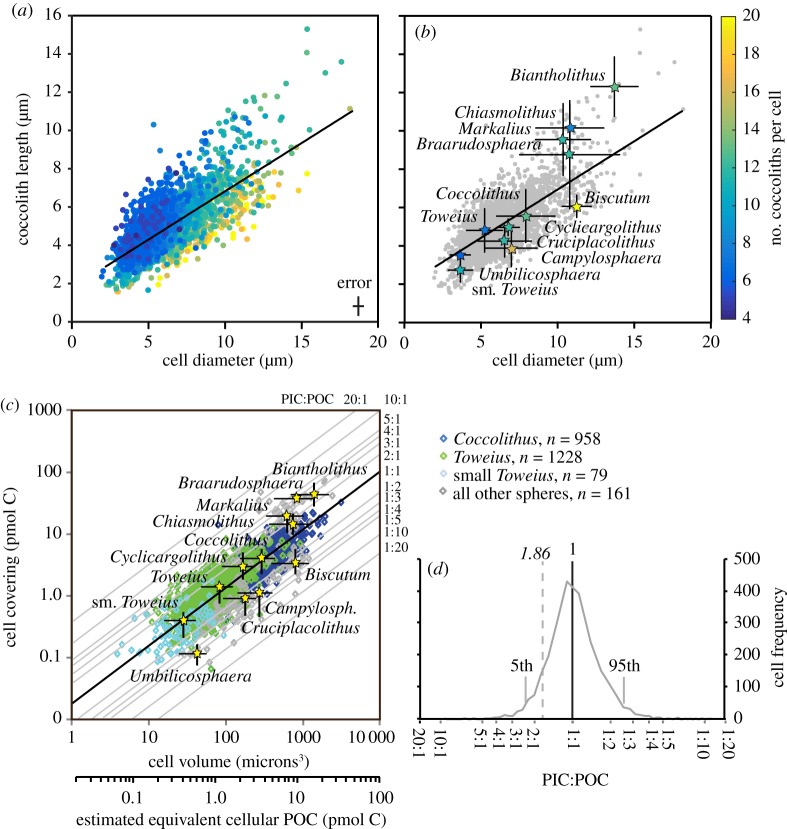
Coccosphere biometric data and calculated PIC and POC per cell for 2,426 PETM coccospheres. In (*a*) are plotted cell diameter (*Θ*) against coccolith length (*C*_L_) for each coccosphere, and the number of coccoliths per coccosphere (*C*_N_) indicated by the colour. The cell diameter of *Biscutum* are the diameters of spherical equivalents (because *Biscutum* cells are ovoid to cigar-shaped). In (*b*), the same data are shown with the means of each taxon highlighted (stars) and the 5–95% spread of the taxon size data indicated by the black bars. The colour of the star indicates average *C*_N_. A broad correlation between coccosphere *Θ* and *C*_L_ is a persistent feature of coccosphere geometry (linear trend-line in *a* and *b*), with larger coccospheres typically associated with larger coccoliths. The notable scatter in the relationship between *Θ* and *C*_L_ is a result of varying *C*_N_. In (*c*), all coccospheres have been converted into cell volume and estimated POC per cell and *C*_L_ and *C*_N_ have been combined to calculate PIC per cell. Diagonal lines indicate PIC to POC ratios. In (*d*) the PIC:POC of the coccospheres are shown as a frequency plot with the 5th and 95th percentiles indicated and the theoretical ratio of 1.86 where photosynthesis balances calcification resulting in no net carbon fixation [21]. The error/uncertainty black bars in (*c*) are the cumulative highest and lowest PIC and POC values we can calculate using all the uncertainties/errors listed below. Errors/uncertainties include direct measurements of *Θ* from the coccospheres (minimal measurement error with high reproducibility). Uncertainty with the counting *C*_N_ is minimal for low coccolith number—up to 9—increasing as the *C*_N_ increases, up to an uncertainly of approximately ±2 at a *C*_N_ around 18 upwards [23], resulting in under or overestimate in PIC of approximately ±7% to approximately ±10%. Uncertainty associated with converting inner coccolith cycle (the parameter we can measure accurately on the coccospheres) to total *C*_L_ (which is obscured by coccolith overlap) is approximately 15%. Uncertainty associated with application of shape factors suggested by ref. [32] to be approximately 20%. Uncertainties in estimating POC from errors in *Θ* measurement are as above and uncertainty in the ref. [38] equation uses the published 95% confidence intervals.

The coccospheres we have imaged and measured from a range of sites provide us with accurate taxon-specific geometry characteristics, i.e. the fundamental coccosphere traits of how coccoliths surround each cell and the cell-to-coccolith size relationship, as well as the frequency distributions of those relationships. The deviation of taxon averages from the overall trend line between cell size and coccolith size illustrates the taxon-specific ranges in geometries for our Palaeogene nannofossils, which are principally a function of varying numbers and degrees of overlap of coccoliths on the coccospheres (figure 4*a*,*b*). Those taxa that lie below the trend line have either high numbers of small coccoliths per cell (e.g. *Biscutum*) or relatively low levels of coccolith overlap on their coccospheres (e.g. *Umbilicosphaera*) (figure 4*b*). Correspondingly, the taxa that lie above the trend line have fewer, larger coccoliths for a given cell size (e.g. *Chiasmolithus*) or greater coccolith overlap (e.g. *Biantholithus*) (figure 4*b*). These subtle differences in the relationships between *C*_N_, *C*_L_ and *Θ* give rise to the taxon-specific power relationships (table 1). Converted to cellular PIC and POC, these coccosphere data reveal that most cells lie at, or close to, a PIC:POC ratio of 1 (70% fall between 0.5 and 1.5), irrespective of cell size (figure 4*c*,*d*). Some taxa show averages that lie away from this line, for example, relatively lightly calcified forms with lower PIC:POC (e.g. *Campylosphaera* and *Biscutum)* lie below the 1 : 1 line, with ratios down to 0.06 (approximately 1 : 17), and more heavily calcified taxa (e.g. *Braarudosphaera* and *Biantholithus)* lie above the line with cells achieving, in some instances, unusually high PIC:POC ratios of up to 14. The similarity in position of taxon averages between figure 4*b* and 4*c* is in part because the POC axis and cell-size axis are recording essentially the same parameter, as our calculation of POC is based on cell volume [38]. But the position of the coccospheres on the *C*_L_ and PIC axes shows that taxa with fewer, larger coccoliths tend to have higher PIC:POC than those with greater numbers of smaller coccoliths. Because of this, the murolith taxa, with larger reconstructed cell sizes and higher numbers of smaller coccoliths, fall below the 1 : 1 line, averaging PIC:POC of between 0.8 and 0.36 (equivalent to a maximum of 1 : 3). Likewise, although the individual nannoliths of *Fasciculithus* and *Sphenolithus* are relatively heavily calcified, their high numbers on large cells result in estimated PIC:POC that is lower than might have been supposed, still close to the 1 : 1 line (PIC:POC estimates of 1.2–1.3).

**Figure 5.**
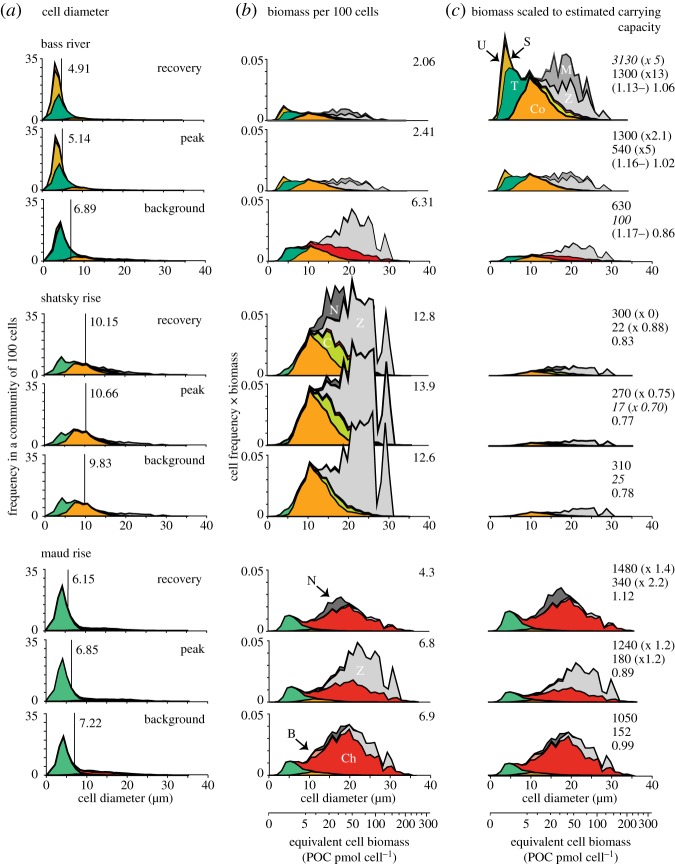
Reconstructed cellular communities and their biomass across three time-slices of the PETM—the pre-CIE (labelled background), the onset to peak of the CIE (labelled peak), and the recovery—from Bass River, Shatsky Rise (ODP Site 1209) and Maud Rise (ODP Site 690). In (*a*) are size frequency histograms of cell diameter (of 100 cells) with mean cell diameter noted in the vertical black line and value, separated into taxon by colour and with a prefix: U—*Umbilicospheara*, S—small *Toweius*, T—*Toweius*, Co—*Coccolithus*, C—*Campylosphaera* and *Cruciplacolithus*, Ch—*Chiasmolithus*, B—*Biscutum*. In grey are the cell diameters for taxon that do not preserve as coccospheres: N—nannoliths (including *Discoaster*, *Sphenolithus* and *Fasciculithus*), M—muroliths, and Z—*Zyghrablithus*. In (*b*), cell diameter has been converted into cell biomass with the total area under the curves equalling total cell biomass and the distance along the *x* axis indicating how that biomass is distributed according to cell size. The value given is the total biomass of 100 cells. In (*c*), biomass from (*b*) has been scaled according to estimated changes in nutrient availability at each site and across the event. Again, the area under the curve corresponds to total biomass. The values on the right give amount of (pmol) POC per millilitre (top), number of cells per millilitre (middle), and community PIC:POC (the total PIC of 100 cells divided by the total POC of 100 cells, bottom). For the PIC:POC, in brackets is the PIC:POC of a ‘bloom’ end-member community dominated by a low diversity mesotrophic-eutrophic subset of the community. In brackets for the other two values are the times change from pre-CIE values. The values that are italicized indicate which values were used as tie-points in figure 3. For Bass River, the time-slices include data averaged across 358.94–357.56, 357.38–356.83, and 352.59–349.03 mbs; for Site 1209 across 216.80–216.36, 216.35–216.22, and 216.17–215.47 mcd, and for Site 690 across 172.33–171.43, 170.61–170.21, and 169.40–167.14 mbsf.

The integration of cell sizes and calculated PIC and POC in our cellular community reconstructions illustrates that the size distributions across Palaeogene communities are strongly biased towards relatively small cell sizes, with modal cell diameters of 4–5 µm at all sites, although there is a broader cell-size range at open-ocean Shatsky Rise (figure 5*a*). Superficially, all three sites appear to show little variation in cell size distribution through time with no significant changes standing out. However, cell diameter hides the fact that larger cells, even if at low abundance, volumetrically outweigh smaller cells and this is clearly the case when we transform cell diameter data into cell biomass. This first set of biomass plots (figure 5*b*) illustrates the disproportionate contribution of the rarer but larger cells in the distribution of biomass across the cell-size range at a constant abundance. The impact of large cells is particularly prominent at Maud Rise, where the presence of *Chiasmolithus* results in the majority of community biomass packaged into a few, large cells. Likewise, at Bass River and Shatsky Rise, we see a significantly more balanced distribution of biomass across the community than is suggested by the cell diameters alone.

Across the time-slices at Shatsky Rise and Maud Rise, the community biomass structure of 100 cells remains relatively similar (total biomass shows little change), even though the taxa contributing biomass change significantly. For example, at Maud Rise, *Chiasmolithus* and *Zygrhablithus* show a large shift in abundance between the pre-CIE and peak-CIE time-slices but this has little effect on the overall biomass distribution across cell sizes. A greater difference through time is seen at Bass River, where biomass associated with larger cells declines in the PETM peak and recovery resulting in a greater than halving of total biomass per 100 cells. These plots with constant cell number are useful because they graphically illustrate how different the communities are between the sites in terms of how biomass is packaged across cells. However, they provide no information on variations in standing stock from site to site. Therefore, in figure 5*c* we have scaled the biomass plots according to estimated nutrient availability for each site and for each time-slice. The Bass River and Maud Rise communities show higher levels of inferred biomass per unit seawater than Shatsky Rise, up to at least 10 times higher in the recovery interval. Bass River shows a marked increase in total biomass through the time-slices, which was part of our initial assumptions underlying figure 3. However, because of the shift in community cell size structure, this increase in biomass is accompanied by a huge increase in cell numbers, with a conservative estimate of a 13-fold increase (up to 1300 cells ml^−1^) from pre-CIE values. This influx of small eutrophs (*Toweius*) is consistent with other evidence for an increase in productivity at Bass River, including total inorganic carbon accumulation [26] and biogenic magnetite [39]. If the productivity was strongly seasonal, as it may have been at Bass River where runoff was dependent on seasonal variations in precipitation [41], then these levels of cell numbers could certainly have approached a reasonable definition of a ‘bloom’. If blooms did occur (see further discussion below), the communities were likely dominated by a mesotroph-eutroph subset of the assemblage, particularly *Toweius*, a scenario that is analogous to modern seasonal, nutrient-driven blooms of eutrophs in the related family Noelaerhabdaceae (e.g. *Emiliania* and *Gephyrocapsa*), which can reach in excess of a million cells per litre during a bloom [41,42].

A very different picture emerges at Shatsky Rise, a Pacific gyre site, where baseline standing stocks of cells would have been initially low and declined further at the peak of the PETM. Although similar overall size distributions were maintained throughout the event, this resulted in proportionally fewer cells when community biomass declined. The overall cell size character of the Shatsky Rise communities was very different to Bass River, with higher relative abundances of oligotrophs, such as *Discoaster* (and other nannoliths), and mesotrophs dominated by the large species *Coccolithus pelagicus*. A greater proportion of the POC was therefore packaged into larger cells. The same is true of the off-shelf site at Maud Rise where an increase in the large mesotrophic *Chiasmolithus* results in higher average cell-size and biomass. The Maud Rise communities do show some degree of change in cell-size distribution through the event, with an increase in cell numbers and a shift towards small-celled mesotrophs during the recovery phase, but not to the same extent as is seen at Bass River. The overall biomass estimates scaled to POC are, however, likely an overestimate at Maud Rise, with the higher PI values skewed because of temperature effects on the index.

The overall PIC:POC of these communities varies little across the event and between sites (figure 5). Bass River shows an increase from 0.86 to 1.06, reflecting a decline in contribution from lower PIC:POC nannoliths and holococcoliths in the community. The PIC:POC of just the *Toweius* taxa (shown in brackets in figure 5) illustrates a possible end-member bloom scenario. The Shatsky Rise community PIC:POC values are lower throughout the event (0.77–0.83) because of the persistent contribution of nannoliths, muroliths and holococcoliths, and the placoliths *Campylosphaera* and *Cruciplacolithus*. Maud Rise shows the largest change in PIC, from 0.89 to 1.12, mainly because of the increase in *Chiasmolithus*.

## 4. Discussion

### (a) Calcareous nannoplankton productivity, calcification and climate feedbacks

It is likely that increased sequestration of carbon was a critical sink for high CO_2_ across the PETM, in addition to the silicate weathering feedback that alone cannot account for the rapidity of climate recovery [1,2]. What remains uncertain is the extent to which calcareous nannoplankton productivity, export and burial influenced this process. Calcareous nannoplankton contribute both to the organic carbon pump and the carbonate counter pump and therefore play a multifarious role in carbon sequestration (e.g. [16–21]). They provide a direct, long-term sink for carbon through the production and burial of organic and inorganic matter and indirectly influence carbon export through the provision of ballasting minerals [17,19]. However, variations in coccolithophore calcification have been implicated in shorter-term surface water CO_2_ buffering, because calcification reduces the rate at which the surface ocean can absorb atmospheric CO_2_—the ‘CO_2_-calcification’ feedback [18,19].

Our new data provide additional dimensions to the assessment of nannoplankton response and function at the PETM, allowing us to assess potential shifts in PIC:POC, cell-size distribution and biomass production. In the sections below we consider the potential influence of these combined factors on the production and export of carbon to the deep sea and hence the sign and strength of the resulting climate feedbacks.

### (b) The role of nannoplankton PIC:POC in surface ocean buffering—the ‘CO_2_-calcification’ feedback

A CO_2_-driven reduction in coccolithophore calcification and hence increase in buffering capacity of surface waters would constitute a negative feedback to rising atmospheric CO_2_ and *vice versa*, although the reality/magnitude of these feedbacks are contentious with a range of responses reported from culture experiments and the fossil record [19]. Culture manipulation experiments focusing on a limited number of extant taxa have largely, but not exclusively, reported reduced calcification under elevated CO_2_ treatments, leading to a negative CO_2_-calcification feedback hypothesis [16,19,43]. However, the opposite has also been argued, with [21] suggesting that increased coccolith calcification during high CO_2_ intervals at glacial terminations may have constituted a positive feedback to increasing CO_2(atm)_ on millennial timescales. At the PETM, while there are significant migration and population shifts across the event [7,8,44], there is little evidence for direct effects on calcification from surface water acidification [2,24] and therefore little obvious CO_2_-calcification feedback. There are reports of morphologically-modified liths in one or two taxa (e.g. *Discoaster*, [14]) and minor changes in coccolith thickness [24], but these would have had little effect on the amounts of calcite being produced by these taxa, and overall the total nannoplankton population appears to have been little affected [15,44,45].

Our documentation of the PIC:POC character of the PETM nannoplankton community is the most comprehensive to date of its kind, and outstrips even our knowledge of modern coccolithophores. We have PIC:POC estimates for approximately 20 taxa from coccosphere observations and for nine taxa from coccosphere reconstructions, representing the majority of PETM diversity. The overwhelming outcome of this compilation is that the PIC:POC of most species is similar, with most lying within a narrow range from 0.5 to 1.5. The dominant taxa all have very similar PIC:POC with the more extreme end-member taxa limited to rare and/or sporadic occurrences (figure 4*c*,*d*). Therefore, even though our reconstructions of nannoplankton biomass across the PETM include evidence of significantly large changes, these do not translate into significant community PIC:POC variation, either through the time interval or even between our high-productivity and oligotrophic end-member sites. Overall, there is little scope for significant shifts in community PIC:POC unless the communities transitioned towards unrealistic species compositions; such as ones dominated by, for example, murolith-bearing coccolithophores (giving rise to lower PIC:POC) or *Biantholithus* or *Braarudosphaera* (giving rise to much higher PIC:POC). In reality, most sites at this time, regardless of latitude or nutrient regime, are dominated by varying proportions of *Toweius* and *Coccolithus* [44], both of which have similar cellular PIC:POC (despite different cellular levels of PIC and POC), with some degree of contribution by other placoliths, murolith and nannolith-bearing species. Our observations also indicate that shifts in community PIC:POC do not remotely approach the ratio of 1.86 where the balance of photosynthesis and calcification, in the most extreme scenario, results in no net carbon fixation [21]. The only other attempt to reconstruct coccolithophore PIC:POC through time reported similarly small variations across two Quaternary glacial termination events [21]. It is therefore hard to envisage how such small changes in PIC:POC could significantly alter overall nannoplankton calcite production and any minor variations in the amounts being produced per cell would be far outweighed by changes in species growth rates [24,29] or overall productivity. Given the narrow confines of nannoplankton PIC:POC diversity, the most significant influence on the rain ratio and associated buffering capacity would come from varying the ratio of calcifying to all non-calcifying plankton, rather than any change within the nannoplankton themselves. For comparison, modern-day-equivalent total surface-water PIC:POC values, i.e. coccolithophores plus everything else, range from 0.001 to 0.4 [41,46], illustrating how small the coccolithophore inorganic carbon contribution can be, even taking into account the differences in plankton make-up between Palaeogene oceans and today. Therefore, while tendencies towards either more heavily/lightly calcified genotypes or species [47,48] may well be a response to seawater chemistry drivers, the hypothesis that the PIC:POC of nannoplankton/coccolithophores itself represents a significant feedback in the buffering capacity of the surface ocean appears to be a red herring.

### (c) Nannoplankton cell size, biomass and export—the productivity feedback

The burial of organic and inorganic carbon in shelf environments increased by an order of magnitude across the PETM [26,49] amplifying the carbonate burial response to silicate weathering, and delivering a necessary additional sink for excess atmospheric CO_2_. This increased burial of carbon, at least in part, could have resulted from higher levels of total plankton production and export [3], including calcareous nannoplankton, fuelled by increased runoff and nutrient supply [40]. The nannoplankton abundance data at Bass River confirm a shift to assemblages indicative of higher productivity but our community cell size record highlights the fundamental shift in population character, with a dramatic shift to smaller cell sizes and many more cells, equivalent in estimated cell numbers to modern bloom conditions.

The controls on plankton export are complex but could this shift in biomass packaging (towards smaller cells) further enhance the productivity feedback over and above the increased numbers, for example, by changing the effects of grazing, remineralization, or ballasting? On the one hand, smaller cells could be more easily recycled in surface waters, as well as being less effective ballasting agents. However, more importantly, there are significantly more cells being produced, increasing the likelihood of collision and aggregate formation, and hence enhancing carbon export/transfer efficiency [50,51]. Similarly, if these elevated cell numbers are associated with seasonally intensified bloom concentrations then this increase in biomass would promote greater levels of grazing (and increase contact/encounter rates) and the formation of faecal pellets that result in higher levels of transfer efficiency and less recycling.

### (d) Evidence for changing export and transfer efficiency

**Figure 6.**
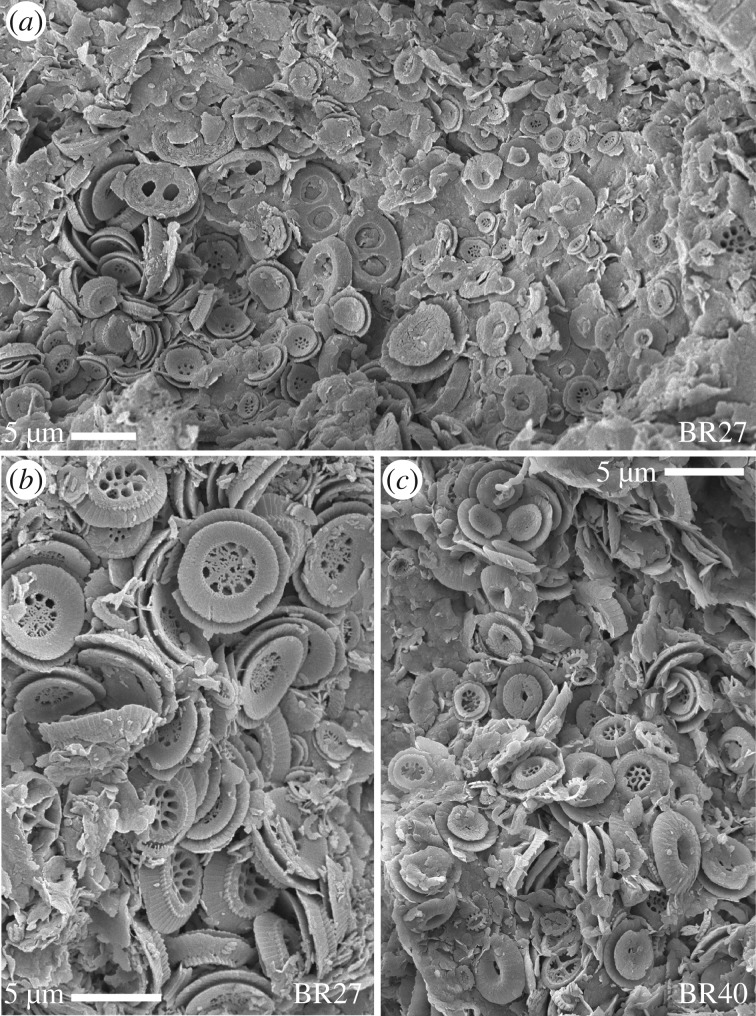
Scanning electron micrographs of fresh rock surfaces from the PETM recovery interval of Bass River with concentrations of coccoliths (mainly small and larger *Toweius*, including collapsed coccospheres), which represent aggregates or faecal pellets providing snapshots of the surface water populations. (*a*) and (*b*) are from two aggregations in sample BR27 (349.82 mbs) and (*c*) is an aggregation in sample BR40 (352.49 mbs).

It is difficult to unequivocally demonstrate the occurrence of bloom events in geological successions because the fossil record is typically time-averaged and so incapable of capturing very short-term phenomena (days to weeks). The exception to this are atypical laminated deposits that continuously record depositional processes and which remain undisturbed by mixing and/or bioturbation due to quiescent and hypoxic conditions. These exceptional sediments preserve records of exported aggregates and faecal pellets that are rich in microfossils (especially nannoplankton and diatoms) and in some cases the extent of these concentrations are considered to represent the fallout from bloom events (e.g. [31,52–54]). Although none of our successions are laminated in nature, both the Bass River and Lodo sediments are sufficiently undisturbed to reveal concentrations of nannoplankton that must represent exported aggregates and faecal pellets. In the case of Bass River, in particular, the coccolith concentrations are low diversity or monospecific and always dominated by *Toweius* species (figure 6), precisely as would be expected if these were blooms. This type of preservation is rare and often discontinuous (largely dependent on levels of bioturbation/oxygenation) and so we have been unable to systematically collect observations that provide comparative information throughout the PETM and across the different sites. However, the bulk of our faecal pellet/aggregate observations do come from the recovery interval of the PETM confirming that at this time there were low diversity assemblages, dominated by the principal eutroph group, *Toweius*, and exported to the seafloor in relatively large packages, indicative of effective transfer efficiency.

Away from the shelf sites, there is evidence of a range of potential biological influence on carbon sequestration, dependent on oceanographic setting. Maud Rise appears to represent an intermediate productivity state between the shelf and open-ocean sites, and our cell size and biomass data would suggest a trend towards increasing biomass into the PETM event and a shift towards small-celled mesotrophs during the recovery phase. This shift towards the small mesotrophs is not as significant as that seen at Bass River, but nevertheless could be a biological amplifier to carbonate accumulation and is supported at Maud Rise by high nannofossil carbonate accumulation rates, perhaps again associated with increased runoff during the recovery phase [55].

At open-ocean Shatsky Rise, we see no evidence of increases in nannoplankton production and no major changes in cell size distribution during the recovery interval. Rather, the assemblage data here reveal greater proportions of oligotrophs [7] reflecting increased stratification and expansion of oligotrophic areas across the PETM [3,8,56]. In these settings it appears unlikely that nannoplankton primary production had any direct feedback-role on increased seafloor carbonate accumulation and that this was instead the result of enhanced calcite preservation as the calcite compensation depth (CCD) shoaled and ocean carbonate saturation increased, as is widely observed and modelled (e.g. [57,58]). Increased carbonate production could have accentuated CCD shoaling or contributed to higher accumulation rates above the CCD, but we think neither are likely given the minor changes in nannoplankton production and community cell-size structure, mirrored in the overall low estimates of export productivity in open ocean areas in general [3].

## 5. Summary and conclusion

Our fossil coccosphere geometry data provide new insights into the cellular-level response of this dominant plankton group during the PETM global warming event. We have been able to reconstruct the evolving cell-size distribution, biomass partitioning, and biomass and inorganic carbon yield of three contrasting fossil calcareous nannoplankton communities (shelf, off-shelf and open ocean) through the PETM onset and recovery, revealing distinctly different responses at each, consistent with previous palaeoecological data, and providing support for a significant plankton productivity feedback at this time. We document differences in numbers of cells and levels of cellular PIC and POC produced at our different sites, with the most dramatic changes across the PETM at the palaeo-shelf Bass River location. During the recovery interval, the Bass River nannoplankton community underwent a major shift towards increased production of smaller cells. However, despite these large changes in taxic composition and community structure, we see little significant difference in fundamental cellular PIC:POC, reflecting the underlying observation that the PIC:POC of most nannoplankton species is very similar. Given these results we consider the idea that nannoplankton PIC:POC might be a major control on surface water buffering and CO_2_ drawdown, to be a red herring. Nevertheless, the cellular abundance and cell size changes likely modified food chain structure and export efficiency, providing a link between calcareous nannoplankton productivity, carbon sequestration and climate recovery. While the ultimate burial of carbon is what is fundamental to CO_2_ removal and climate regulation, we need to further examine the potential implications of how calcareous nannoplankton/coccolithophore PIC and POC is packaged (how much and in what size cells) for ballasting and grazing and hence carbon export and export efficiency.

## Supplementary Material

Detailed methodology for utilising coccospheres and producing reconstructions

## Data accessibility

The datasets supporting this article are available via the National Geoscience Data Centre.
