# Supplementary material for: Warm plankton soup and red herrings: calcareous nannoplankton cellular communities and the Palaeocene–Eocene Thermal Maximum
Source: Philos Trans A Math Phys Eng Sci. 2018 Sep 3;376(2130):20170075. doi: 10.1098/rsta.2017.0075 (PMC6127380; doi:10.1098/rsta.2017.0075)
Supplement: Detailed methodology for utilising coccospheres and producing reconstructions [file rsta20170075supp1.docx]

Philosophical Transactions A doi:10.1098/rsta.2017.0075

**Warm plankton soup and red herrings:**

**Calcareous nannoplankton cellular communities and the Palaeocene-Eocene Thermal Maximum**

Samantha J. Gibbs, Rosie M. Sheward, Paul R. Bown, Alex J. Poulton and Sarah A. Alvarez

Supplementary Material

Direct measurements and coccosphere reconstructions

***Placoliths and Braarudosphaera***

The PETM placolith coccolithophore data were simplified into seven, broadly genus-level groups: *Biscutum* (monospecific, *B. bralowerii*), *Chiasmolithus* spp. (several species at most), *Coccolithus* (monospecific, *C. pelagicus*), *Cruciplacolithus* spp. + *Campylosphaera* spp. (several species at most), *Umbilicosphaera* (monospecific *U. bramlettei*), and *Toweius* (divided into *Toweius* spp. and very small *Toweius* spp., <2 microns). For each group, we determined the taxon-specific power relationship between C_N_, C_L_ and Θ (Table 1), where knowledge of any two parameters enables calculation of the third [Gibbs et al., 2013]. PIC per cell was estimated using C_N_ and an estimated mass per coccolith using integrated coccolith dimension data and taxon-specific conversion factors [Sheward et al., 2017; Young and Ziveri, 2000], modified for extinct taxon (Table 1). *Braarudosphera* was straightforward, as both modern and fossil coccospheres form a perfect dodecahedron and the 12 nannoliths are simple pentagons forming an imperforate covering around the cell. *Braarudosphaera* nannolith thickness reflects the range seen in both SEM and LM images of coccospheres and disarticulated nannoliths. Estimated POC per cell is based on cell size, assuming the proportional scaling of cellular organic carbon content with cell volume (*V*) using the relationship derived by [Menden-Deuer and Lessard, 2000] for protists, excluding diatoms:

$${log}_{10}POC \left( pg {cell}^{-1} \right)= {log}_{10}(-0.665\pm0.132)+ 0.939\pm0.041 {log}_{10}V$$

***Muroliths and Holococcoliths***

For these taxa without preserved PETM coccospheres, we have estimated C_N_ and Θ based on curvature of proximal surfaces and angle of outer wall. Further supporting evidence is provided by rare, partially intact fossil coccospheres, the numbers of coccoliths associated with collapsed fossil coccospheres, and the numbers of coccoliths per cell in modern analogue species. In comparison to placolith-bearing taxa, modern murolith- and holococcolith-bearing coccolithophores typically have higher numbers of coccoliths per cell and therefore larger cell sizes relative to coccolith size. Our PIC estimates use coccolith volume calculations based on simplified coccolith morphologies multiplied by estimated C_N_. For muroliths, the individual coccolith mass was estimated using a three-dimensional ellipse accounting for the slope of the coccolith walls and removing varying amounts of mass, dependent on the number and size of holes present across the murolith (Table 1). A similar approach was used for the *Zygrhablithus bijugatus* holococcolith using a mass estimate based on a cruciform spine structure attached to a simple elliptical murolith with a hollow central cavity. POC estimates were calculated from estimated Θ, as above.

***Nannoliths***

We have used the morphology of individual nannoliths to reconstruct cell size and number of nannoliths per cell, in particular the curvature of *Discoaster* nannoliths and curvature of proximal surfaces of *Fasciculithus* and *Sphenolithus* nannoliths. *Fasciculithus* and *Sphenolithus* have relatively shallow proximal curvature and we therefore assume they are similar to the muroliths in having large numbers of nannoliths per cell and a correspondingly large cell size relative to the nannolith size. This assumption has also underpinned previous reconstructions of these taxa (e.g., Towe, 1979). For discoasters, we applied a reconstruction that uses a rosette-shaped morphology, because this morphology dominates (50-85%, *D. multiradiatus* and *D. salisburgensis)* in these communities. The rosette nannoliths have a clear, albeit low, degree of curvature, allowing for assumptions about the relative size of the cell that these nannoliths surrounded. We applied some moderate nannolith overlap, assuming the nannoliths would form a near-continuous protective cell covering, in effect producing a reconstructed coccosphere similar to modern *Coccolithus* or *Calcidiscus* in terms of number of nannoliths and degree of nannolith overlap [Young et al., 2017]. For calculating PIC, discoasters were treated as a disc of given thickness multiplied by our estimate of number of nannoliths per cell (Table 1). Sphenoliths and fasciculiths were treated as cones with concave proximal surfaces and varying degrees of hollow embayments or pits as seen in SEM observations of exceptionally well-preserved examples [Young et al., 2017]. POC estimates were calculated from estimated Θ, as above.

The greatest uncertainty in the overall community reconstructions comes with estimates of discoaster cell size and PIC:POC. Our discoaster coccosphere reconstructions lie at the smaller end of their potential cell size range, as we estimate relatively low lith numbers and a moderate degree of overlap. Our conservative cell size estimates may therefore represent a high PIC:POC end member, because smaller spherical cells have higher PIC:POC than larger spherical cells of the same taxon. This is significant because, although discoasters are rare in our communities (e.g., 0.76 –4.7% cell abundance at Bass River), their relatively large cell sizes (even using conservative, small end-members) result in a disproportionate biomass (15-28%). Because of this, any under-estimate of discoaster cell size and over-estimate of PIC:POC could have a significant impact on our biomass and resultant community PIC:POC, pushing biomass towards an even more exaggerated distribution bias towards larger cells, and a resultant lower community PIC:POC.

***References***

Gibbs SJ *et al.* 2013 Species-specific growth response of coccolithophores to Palaeocene–Eocene environmental change. *Nat. Geosci.* **6**, 218–222. (doi:10.1038/ngeo1719)

Menden-Deuer S, Lessard EJ. 2000 Carbon to volume relationships for dinoflagellates, diatoms, and other protist plankton. *Limnol. Oceanogr.* **45**, 569–579.

Sheward RM, Poulton AJ, Gibbs SJ, Daniels CJ, Bown PR. 2017 Physiology regulates the relationship between coccosphere geometry and growth phase in coccolithophores. *Biogeosciences* **14**, 1493–1509. (doi:10.5194/bg-14-1493-2017)

Towe KM. 1979 Variation and systematics in calcareous nannofossils of the genus Sphenolithus. *Am. Zool.* **19**, 555–572.

Young JR, Ziveri P. 2000 Calculation of coccolith volume and it use in calibration of carbonate flux estimates. *Deep Sea Res. Part II Top. Stud. Oceanogr.* **47**, 1679–1700. (doi:10.1016/S0967-0645(00)00003-5)

Young JR, Bown PR, Lees JA. 2017 Nannotax3 website. See http://www.mikrotax.org/Nannotax3/ (accessed on 9 January 2017).
